# Supplementary material for: Retrospective analysis of estrogen receptor (ER), progesterone receptor (PR), human epidermal growth factor receptor-2 (HER2), Ki67 changes and their clinical significance between primary breast cancer and metastatic tumors
Source: PeerJ. 2024 May 15;12:e17377. doi: 10.7717/peerj.17377 (PMC11102064; doi:10.7717/peerj.17377)
Supplement: Supplemental Information 1 [file peerj-12-17377-s001.pdf]

STROBE Statement—Checklist of items that should be included in reports of ***cross-sectional studies***

	Item No	Recommendation
**Title and abstract**	1	(*a*) Retrospective analysis of ER、PR、HER2、Ki67 changes and their clinical significance between primary breast cancer and metastatic tumors
		(*b*) Objective. To explore the relationship between receptor heterogeneity and clinicopathological characteristics in 166 patients with invasive breast cancer during metastasis.<br>Methods. We conducted a retrospective analysis of 166 patients diagnosed with metastatic breast cancer through biopsy, who were admitted to our hospital from January 2018 to December 2022. Statistical analysis was employed to assess the heterogeneity of receptors in both primary and metastatic lesions, including estrogen receptor (ER), progesterone receptor (PR), human epidermal growth factor receptor-2 (HER2), Ki67, as well as their correlation with clinicopathological features such as tumor size, lymph node metastasis, treatment regimen, and disease-free survival.<br>Results. The discordant expression rates of ER, PR, HER2, Ki-67 and Luminal classification between primary and metastatic lesions were 21.7%, 41.6%, 8.9%, 34.4% and 36.8%, respectively. The median DFS for primary HER2(-) to metastatic HER2(+) was 96 months, which was relatively high. The Cox multivariate regression analysis revealed that the expression differences of ER, PR, HER2, and Ki67 were not influenced by endocrine therapy and chemotherapy. However, a statistically significant difference in HER2 expression was observed with targeted therapy. Tumor size was correlated with ER and Ki67 receptor status ($P = 0.019, 0.016$). Tumor size was not correlated with PR, and HER2 ($P = 0.679, 0.440$). Lymph node metastasis was not associated with changes in ER, PR, HER2, and Ki67. The discordant rates of ER, PR, HER2, and Ki-67 in patients with local recurrence were 22%, 23.7%, 5.1%, and 28.8% respectively, whereas those in patients with distant metastasis were 21.5%, 36.4%, 10.3%, and 31.8% respectively.<br>Conclusions. The expression levels of ER, PR, HER2, and Ki-67 in primary and metastatic breast cancer exhibit heterogeneity, which is closely associated with the prognosis and treatment outcomes of patients.
**Introduction**		
Background/rationale	2	The incidence and mortality rates of breast cancer are the highest among all female malignant tumors, causing significant harm to women's health. The expression of estrogen receptor (ER), progesterone receptor (PR), human epidermal growth factor receptor 2 (HER-2), and Ki67 is commonly employed in clinical practice to categorize breast cancer subtypes, guiding the selection of appropriate treatment methods. The treatment and prognosis of breast cancer patients are closely correlated with the expression of receptors in the primary lesions. Although the majority of breast cancer patients undergo surgical, endocrine, or chemotherapy interventions, there is still a subset comprising 20-30% who experience metastasis to lymph nodes, chest wall, bone, or liver. There is increasing evidence indicating that approximately 40% of primary and metastatic tumors exhibit altered receptor expression, which necessitates modifications to the treatment plan.

Objectives	3	The present study aims to provide additional clinical references by conducting a retrospective analysis of 166 cases of invasive breast cancer patients, focusing on the heterogeneity in ER, PR, and HER2 expression between primary tumors and metastases, as well as the impact of this variation on prognosis and individualized treatment.
**Methods**		
Study design	4	To explore the relationship between receptor heterogeneity and clinicopathological characteristics in 166 patients with invasive breast cancer during metastasis.
Setting	5	The clinicopathological data of 166 patients with biopsy-confirmed invasive breast cancer metastasis at Qingdao Central Hospital Affiliated to Qingdao University from January 2018 to December 2022 were retrospectively analyzed. The follow-up period commenced on the day of the initial surgical procedure and continued until the occurrence of metastases, encompassing both local recurrence and distant metastasis.
Participants	6	Inclusion criteria: female patients diagnosed with unilateral primary or metastatic breast cancer, who are eligible for surgery or biopsy; Complete clinical and follow-up data were collected. Exclusion criteria: male patients with breast cancer; Patients with bilateral breast cancer; Patients with a history of non-breast malignant tumors; Patients with incomplete clinical and follow-up data.
Variables	7	The analysis included the following variables: gender, age, primary site (left or right), histological type and grade, date of first operation, endocrine therapy, chemotherapy, targeted therapy, EP, PR, HER2 and Ki67 values of the primary site; date and site of metastasis, EP, PR, HER2 and Ki67 of the metastatic lesion. Local recurrence comprised ipsilateral breast, ipsilateral chest wall, and ipsilateral regional lymph node metastases. Distant metastases included contralateral breast, contralateral chest wall, contralateral lymph nodes, bilateral supraclavicular lymph nodes, bone involvement, as well as visceral spread. The follow-up process involved a combination of outpatient reexamination, in-patient reexamination, and telephone follow-up. Disease-free survival (DFS) was defined as the interval between the first operation and the onset of initial metastasis.
Data sources/ measurement	8*	The staining of ER and PR was characterized by the presence of yellow or brown granules in the nucleus. A cell count ≥1% indicated a positive result, while < 1% indicated a negative result. A PR positive rate ≥30% was considered high expression, whereas a PR positive rate < 30% was classified as low expression. HER2 grouping criteria were as follows: HER2 (0), HER2 (1+) and HER2 (2+) without amplification by DISH/FISH were categorized as HER2 negative group; HER2 (2+) with DISH/FISH amplification and HER2 (3+) were classified as HER2 positive group. The Ki67 interpretation criteria involved selecting and counting three or more hot spots of invasive cancer positive cells in high-power fields to determine the average Ki67 index. A Ki67 expression level ≥30% indicated high expression, while <30% indicated low expression.
Bias	9	Not applicable
Study size	10	The clinicopathological data of 166 patients with biopsy-confirmed invasive breast cancer metastasis at Qingdao Central Hospital Affiliated to Qingdao

		University from January 2018 to December 2022 were retrospectively analyzed.
Quantitative variables	11	The staining of ER and PR was characterized by the presence of yellow or brown granules in the nucleus. A cell count ≥1% indicated a positive result, while < 1% indicated a negative result. A PR positive rate ≥30% was considered high expression, whereas a PR positive rate < 30% was classified as low expression. HER2 grouping criteria were as follows: HER2 (0), HER2 (1+) and HER2 (2+) without amplification by DISH/FISH were categorized as HER2 negative group; HER2 (2+) with DISH/FISH amplification and HER2 (3+) were classified as HER2 positive group. The Ki67 interpretation criteria involved selecting and counting three or more hot spots of invasive cancer positive cells in high-power fields to determine the average Ki67 index. A Ki67 expression level ≥30% indicated high expression, while <30% indicated low expression.
Statistical methods	12	(*a*) Descriptive statistics were utilized to summarize the clinicopathological data of patients diagnosed with primary breast cancer and metastatic breast cancer, with the results presented as median values, case numbers, and percentages.
		(*b*) The Kappa consistency test was employed to assess the concordance of ER/PR/HER2/Ki67 between primary and metastatic lesions.
		(*c*) The Kaplan-Meier method was employed for survival analysis, and survival curves were generated. The Log-rank test was utilized to compare the differences in ER, PR, HER2, and Ki67 between the consistent group and the inconsistent group in both primary and metastatic lesions.
		(*d*) A Cox regression model was employed for conducting multivariate analysis of treatment regimen.
		(*e*) The chi-square test was employed to examine the association between recipient status and tumor size or lymph node metastasis. The criterion for statistical significance was set at a level of P<0.05.
**Results**		
Participants	13*	(a) The clinicopathological data of 166 patients with biopsy-confirmed invasive breast cancer metastasis at Qingdao Central Hospital Affiliated to Qingdao University from January 2018 to December 2022 were retrospectively analyzed. A total of 166, 166, 148, and 152 patients had complete ER, PR, HER2, and Ki-67 data for primary and metastatic lesions, respectively.
		(b) Exclusion criteria: male patients with breast cancer; Patients with bilateral breast cancer; Patients with a history of non-breast malignant tumors; Patients with incomplete clinical and follow-up data.
		(c) Not applicable
Descriptive data	14*	(a) The clinicopathological data of 166 patients with biopsy-confirmed invasive breast cancer metastasis at Qingdao Central Hospital Affiliated to Qingdao University from January 2018 to December 2022 were retrospectively analyzed. The analysis included the following variables: gender, age, primary site (left or right), histological type and grade, date of first operation, endocrine therapy, chemotherapy, targeted therapy, EP, PR, HER2 and Ki67 values of the primary site; date and site of metastasis, EP, PR, HER2 and Ki67 of the metastatic lesion. Inclusion criteria: female patients diagnosed with unilateral primary or metastatic breast cancer, who are eligible for surgery or biopsy; Complete clinical and follow-up data were collected.
		(b) Exclusion criteria: male patients with breast cancer; Patients with bilateral

		breast cancer; Patients with a history of non-breast malignant tumors; Patients with incomplete clinical and follow-up data. A total of 166, 166, 148, and 152 patients had complete ER, PR, HER2, and Ki-67 data for primary and metastatic lesions, respectively.
Outcome data	15*	A total of 166 patients met the inclusion criteria. A total of 166, 166, 148, and 152 patients had complete ER, PR, HER2, and Ki-67 data for primary and metastatic lesions, respectively. During a mean follow-up of 59 months (range, 4-204 months), all of the 166 patients (100%) with invasive breast cancer had metastasis, including 59 cases with local metastasis and 107 cases with distant metastasis. Among the patients in this article, 166 cases had complete treatment information for ER, 166 for PR, 157 for HER2, and 148 for Ki67. A total of 158 and 160 patients had complete T stage (tumor size) and N stage (lymph node metastasis). All 166 patients were followed up for a duration ranging from 4 to 201 months.
Main results	16	(*a*) The discordant expression rates of ER, PR, HER2, Ki-67 and Luminal classification between primary and metastatic lesions were 21.7%, 41.6%, 8.9%, 34.4% and 36.8%, respectively. The median DFS for primary HER2(-) to metastatic HER2(+) was 96 months, which was relatively high. The Cox multivariate regression analysis revealed that the expression differences of ER, PR, HER2, and Ki67 were not influenced by endocrine therapy and chemotherapy. However, a statistically significant difference in HER2 expression was observed with targeted therapy. Tumor size was correlated with ER and Ki67 receptor status (P = 0.019, 0.016). Tumor size was not correlated with PR, and HER2 (P = 0.679, 0.440). Lymph node metastasis was not associated with changes in ER, PR, HER2, and Ki67. The discordant rates of ER, PR, HER2, and Ki-67 in patients with local recurrence were 22%, 23.7%, 5.1%, and 28.8% respectively, whereas those in patients with distant metastasis were 21.5%, 36.4%, 10.3%, and 31.8% respectively.
		(*b*) Not applicable
		(*c*) Not applicable
Other analyses	17	Not applicable
**Discussion**		
Key results	18	The expression levels of ER, PR, HER2, and Ki-67 in primary and metastatic breast cancer exhibit heterogeneity, which is closely associated with the prognosis and treatment outcomes of patients.
Limitations	19	The present study, however, is a retrospective analysis with a shorter follow-up time, smaller sample size, and different testing methods. Therefore, further prospective multicenter studies are still needed in the future.
Interpretation	20	In conclusion, this study has demonstrated significant differences in the expression of ER, PR, HER2, and Ki67 between primary and metastatic tumors. These findings have important implications for subsequent treatment planning and prognosis evaluation. Re-biopsy and re-testing of metastatic breast cancer should be considered in clinical practice to facilitate more precise treatment. The present study, however, is a retrospective analysis with a shorter follow-up time, smaller sample size, and different testing methods. Therefore, further prospective multicenter studies are still needed in the future
Generalisability	21	Re-biopsy and re-testing of metastatic breast cancer should be considered in

		clinical practice to facilitate more precise treatment
**Other information**		
Funding	22	This study was supported by Beijing Jingjian Pathology Development Foundation (Grant number: JJDYSG2023-028).

*Give information separately for exposed and unexposed groups.

Note: An Explanation and Elaboration article discusses each checklist item and gives methodological background and published examples of transparent reporting. The STROBE checklist is best used in conjunction with this article (freely available on the Web sites of PLoS Medicine at http://www.plosmedicine.org/, Annals of Internal Medicine at http://www.annals.org/, and Epidemiology at http://www.epidem.com/). Information on the STROBE Initiative is available at www.strobe-statement.org.
